# Composite Materials for Thermal Energy Storage: Enhancing Performance through Microstructures

**DOI:** 10.1002/cssc.201300878

**Published:** 2014-03-03

**Authors:** Zhiwei Ge, Feng Ye, Yulong Ding

**Affiliations:** [a] State Key Laboratory of Multiphase Complex Systems Institute of Process EngineeringChinese Academy of Sciences Beijing 100190 (PR China), Fax: (+86) 010-82544814; [b] University of Chinese Academy of Sciences Chinese Academy of SciencesBeijing 100039 (PR China); [c] Birmingham Centre for Energy Storage Research, University of BirminghamEdgbaston, Birmingham B15 2TT (UK)

**Keywords:** energy transfer, materials science, microstructure, phase-change materials, thermal energy storage

## Abstract

Chemical incompatibility and low thermal conductivity issues of molten-salt-based thermal energy storage materials can be addressed by using microstructured composites. Using a eutectic mixture of lithium and sodium carbonates as molten salt, magnesium oxide as supporting material, and graphite as thermal conductivity enhancer, the microstructural development, chemical compatibility, thermal stability, thermal conductivity, and thermal energy storage performance of composite materials are investigated. The ceramic supporting material is essential for preventing salt leakage and hence provides a solution to the chemical incompatibility issue. The use of graphite gives a significant enhancement on the thermal conductivity of the composite. Analyses suggest that the experimentally observed microstructural development of the composite is associated with the wettability of the salt on the ceramic substrate and that on the thermal conduction enhancer.

## Introduction

Of the global energy budget, 90 % centers around heat conversion, transmission, and storage. A large proportion of this energy is used in the industrial power and processing sectors, which emits large amounts of waste heat due to either low efficiencies as a result of economic and thermodynamic constraints or mismanagement, or both.[1] The waste heat has a wide range of temperatures, from very low-grade (e.g., 30–40 °C in power plants) to very high-grade (e.g. 1600 °C in iron- and steelmaking plants), and is often unsteady and randomly distributed. Effective and efficient utilization of such heat resources requires thermal energy storage. On the other hand, environmental concerns including global warming call for the use of sustainable, renewable energy resources. Among such resources, solar energy is regarded as one of the most promising ones. There are two ways to harvest solar energy: solar thermal and solar photovoltaic (PV). These use energy over the long and short wavelengths of the spectrum, respectively.[2] Thermal energy storage is essential for the solar thermal route due to the intermittent nature of solar radiation. At the heart of these thermal energy storage applications are cost-effective, high-performance storage materials, which form the main motivation of this work.

There are numerous thermal energy storage materials and they can be classified into three types: sensible, latent, and (chemical) reaction heat. This work mainly concerns latent-heat-based storage materials, also called phase-change materials (PCMs). The phase change occurs between liquid and solid states. The most salient features of PCMs are their high energy storage densities and the fact that the phase-change process is isothermal. PCMs can be classified into two categories: organic and inorganic materials.[3] Examples of organic PCMs include paraffin wax, high-chain alkane,[4] fatty acids[5] and fatty acid esters,[6] which are mostly used for applications at *T*<180 °C. Examples of inorganic PCMs include hydrates (mainly for applications at *T*<120 °C) as well as molten salts and metallic materials (for applications at *T*=200–1400 °C). These materials have different characteristics and hence present various issues: metallic materials have a high thermal conductivity but can be corrosive and expensive; inorganic hydrates often suffer from super-cooling and phase segregation; organic PCMs are often flammable and could be toxic in some cases; molten salts can be highly corrosive even for stainless steel; organic PCMs, inorganic hydrates, and molten salts have a low thermal conductivity and hence a low power density due to low heat transfer rates. Various approaches to overcome these limitations have therefore been explored, particularly for organic materials and inorganic hydrates. These include the use of metallic fins and foams[7] and the addition of materials with high thermal conductivity[1, 5, 8] to enhance the property, and encapsulation of the PCMs to reduce/prevent supercooling[9] and leakage issues.[10] However, these approaches cannot be used directly to resolve the issues associated with molten salts for applications at medium to high temperatures. First, metallic materials are prone to irreversible defects arising from hot corrosion at the medium and high temperatures, particularly with molten salts. As a result, metallic foams and fins cannot offer long-term chemical stability. Second, the direct use of materials with high thermal conductivity, particularly carbon, is less effective for inorganic molten salts owing to high surface tension upon melting, leading to poor dispersibility of the materials in the salts and dispersion uniformity.

In this work, we introduce a composite material consisting of a molten salt infused microstructure for medium- and high-temperature thermal energy storage applications. We show that this type of microstructured material is able to resolve the incompatibility of molten salts with carbon materials. It is also possible for the material to have both a high energy density and a high power density.

## Results and Discussion

The PCMs used in this work was an eutectic salt of sodium carbonate and lithium carbonate with a melting temperature of 497 °C and a solid–liquid phase change latent heat of 351 J g^−1^. A ceramic material (MgO) and a carbon material (natural graphite flakes) were chosen as supporting and thermal-enhancement material, respectively. These materials were mixed according to preset mass ratios and shaped into slabs by uniaxial cold compression before sintering in an electric furnace. See the Experimental section for further details.

### Microstructure of the composites

#### Morphological and structural characterization

**Figure 1:**
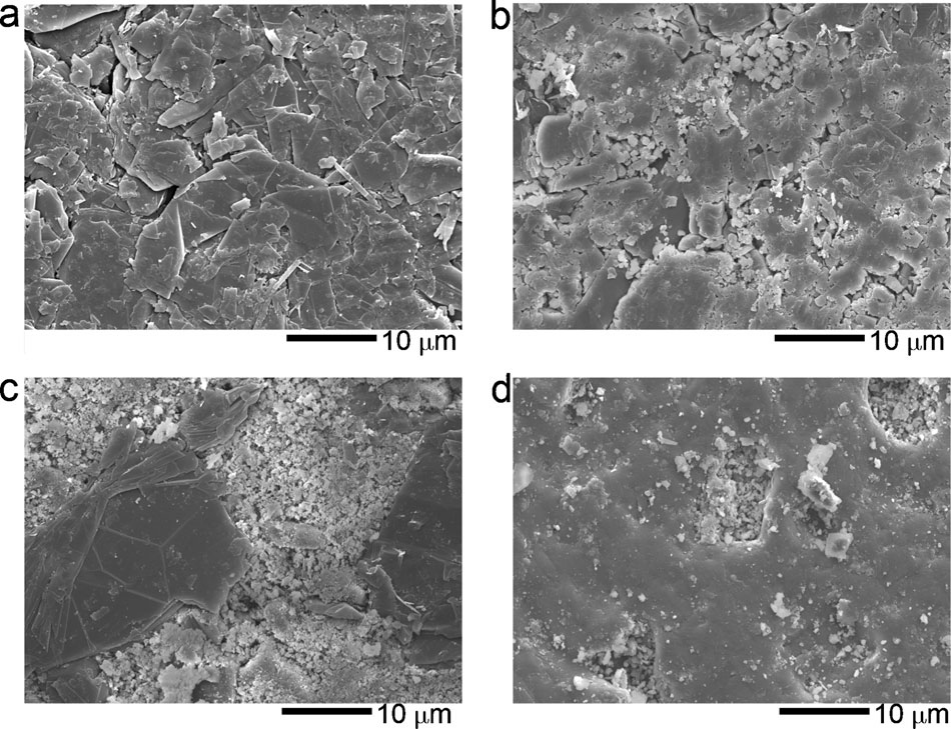
(a) SEM image of green graphite after uniaxial cold compression; (b) SEM image of green composite of graphite–salt after uniaxial cold compression; (c) SEM image of green composite of graphite–salt–MgO with mass ratio of 0.25:1:1; (d) SEM image of sintered composite of graphite–salt–MgO with mass ratio of 0.25:1:1.

Figure 1 (a–c) shows scanning electron microscopy (SEM) images of green slabs made of pure graphite, graphite–salt mixture, and graphite–salt–MgO mixture through uniaxial cold compression, respectively. The pure graphite sample exhibits a layered structure with the two-dimensional surface perpendicular to the direction of compression, as expected. The graphite flakes and salt particles appear to mix well macroscopically but salt-rich regions are apparent, possibly due to significant differences in particle shapes and sizes of the two components. The addition of MgO particles to the graphite–salt mixture leads to graphite-dominant regions separated by salt and MgO particles. Sintering of the slabs changes the morphology significantly, as shown in Figure 1 (d): the graphite- and salt/MgO-rich regions shown in Figure 1 (b) and (c) seem to have disappeared.

**Figure 2:**
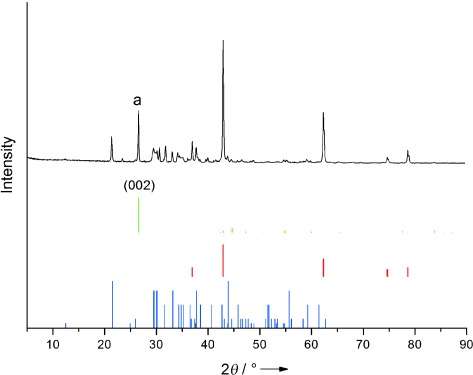
—: XRD patterns of the sintered graphite–salt–MgO composite with a component mass ratio of 0.25:1:1. graphite, MgO, LiNaCO_3_.

**Figure 3:**
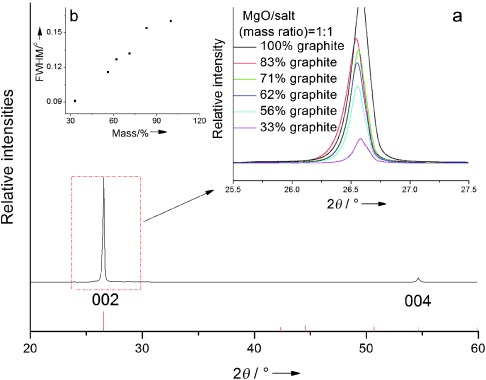
Enlarged XRD patterns of the crystal plan (002) of graphite: a) XRD patterns of composite with different loadings of graphite; b) full width at half maximum (FWHM) obtained from (a).

To illustrate the microstructure development of the composite, X-ray diffraction (XRD) analyses were carried out. Figure 2 shows the crystal phase of the sintered composite containing the graphite, the carbonate salt, and MgO at a mass ratio of 0.25:1:1. The diffraction pattern of the composite contains only the patterns of graphite, MgO, and the eutectic salt, indicating excellent chemical compatibility between the components. The peak of the composite labeled ‘a’ corresponds to the (002) plane of graphite at 2*θ*=26.6°. The graphite flakes consist of a layered structure with interlayers held by van der Waals interactions, giving an interlayer distance of about 3.35 Å. Numerous studies have reported the intercalation of organic or inorganic materials into the interlayers of expanded graphite to prepare graphite composites.[8b, 11] To investigate possible changes of the crystal structure of graphite, the peaks corresponding to the (002) crystal plane of the graphite in different composites are compared in Figure 3 a. One can see a reduction in the relative diffraction intensity due to the decreasing graphite loading in the composites. Unlike the crystal structure of expanded graphite, which has a lower diffraction peak or a larger *d*-spacing than the graphite flakes, the full width at the half maximum (FWHM) of the graphite flakes in the composites decreases with decreasing loading. Such a dependence, as shown in Figure 3 b, is due to the residual strain of graphite after uniaxial cold compression and further constraint of liquefied structure during sintering.

#### Microstructure development of the composite

**Figure 4:**
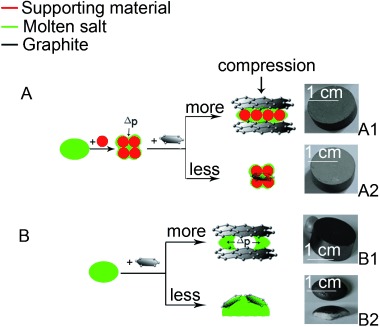
Scheme of microstructure development: Scheme A for graphite–salt–ceramic composites (salt/ceramic mass ratio=1:1); Scheme B for graphite–salt composite.

Based on the above analyses, a proposed microstructure development of the composite materials is illustrated in Figure 4, with the proposed microstructure development for composites consisting of a molten salt, a metal-oxide-based ceramic supporting material, and a carbon material shown in Figure 4 A and that for composites containing a molten salt and a carbon material illustrated in Figure 4 B. The carbon material considered in this work is graphite. In Figure 4, ‘more’ and ‘less’ denote more graphite and less graphite in the composite, respectively, and the four photos are from typical composites corresponding to the indicated conditions.

**Figure 5:**
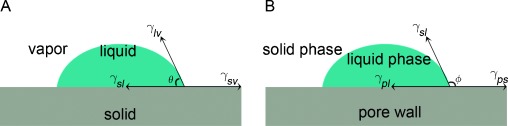
Schematic illustrations of a droplet on a solid substrate: A) Young’s model; B) an analogy of Young’s model for the coexistence of solid and liquid phases in a pore.

First consider a composite comprising salt and ceramic supporting material (Scheme A before graphite is added; see Figure 4 A). The fabrication process involves mixing of the two solid materials. During mixing, pores are created due to interparticle voids. Upon uniaxial cold compression, the interparticle void space is reduced leading to an increase in the green composite density before sintering. During sintering, the salt phase starts to turn into a viscous liquid when the prevailing temperature approaches the melting point, and the composite consists of solid, liquid, and vapor phases. As a result, a significant pressure difference (Δ*P*) is generated, which makes the liquid phase flow into the pores. The pressure difference can be estimated by Δ*P*=2*γ*/*r*, with *γ* being the interfacial energy between the infused liquid molten salt and the solid surface and *r* being the radius of the curvature. According to Young’s equation [Eq. (1)], as illustrated in Figure 5 A:


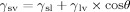
(1)

where *θ* is the contact angle, and the subscripts *s*, *l*, and *v* correspond to the solid, liquid, and vapor phases, respectively. The wettability of the liquid phase on a solid substrate is linked to the contact angle, which determines the direction of the pressure gradient. The ceramic supporting material has a high interfacial energy, resulting in a small contact angle[12] and hence the liquid phase tends to wet and spread on the supporting material surface to displace the solid–vapor interface. Meanwhile, the wetting liquid provides a capillary force to pull the ceramic grains together and rearrange them, facilitating densification of the composite. A progressive grain coarsening and bonding process would occur for the supporting material after the rearrangement and densification, leading to an increase in the rigidity of the microstructure. After sintering, the final structure consists of grains of the supporting material with a solidified liquid network, and possibly some residual pores. Such a rigid microstructure could prevent leaking of the liquid phase during phase change.

Next consider a composite consisting of salt and graphite (Scheme B; see Figure 4 B). The fabrication process is similar to that of the salt–ceramic composite. When the composite temperature approaches the melting point, the solid salt starts to turn into liquid, leading to a pressure difference (Δ*P*). Because graphite has a low interfacial energy, graphite flakes are less likely to be wetted by the liquid molten salt and hence less driving force to displace the solid–vapor interface. The vapor phase in the structure would expand at elevated temperatures. The balance of the above implies that the liquid salt could move outwards, leading to salt leakage and a relatively highly porous composite structure. As a result, graphite–salt composites should only contain a small amount of salt and this has indeed been observed experimentally; see Photo B1 in Figure 4 B where salt leakage is apparent. When the amount of graphite added is very low, the poor wettability of molten salt on graphite means poor dispersion of the particles and hence graphite particles are likely to find their place on the surface of molten salt; see the Photo B2 in Figure 4 B.

Finally, consider the graphite–salt–ceramic composite as illustrated in the Scheme A in Figure 4 A. The vapor phase in the interparticle voids is mostly driven out during the sintering process due to the high interfacial energy of the ceramic material, forming a molten salt liquefied structure (MSLS) of the supporting material with a dense composite structure. The effect of the high interfacial energy of the ceramic material can outweigh that of the low interfacial energy of graphite, preventing salt leakage. These are supported by the two photos (A1 and A2) in Figure 4 A.

### Wettability and thermal conductivity of the composites

**Figure 6:**
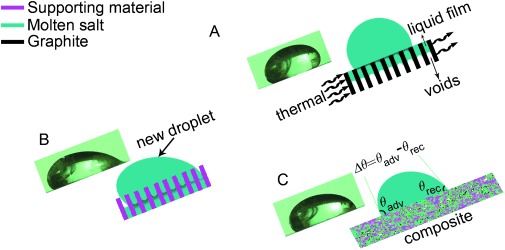
Schematic illustrations of contact-angle hysteresis of a molten salt drop on different substrates, together with corresponding images of the droplets obtained by the high-speed camera: A) Graphite; B) ceramic supporting material; C) graphite–salt–MgO composite.

**Figure 7:**
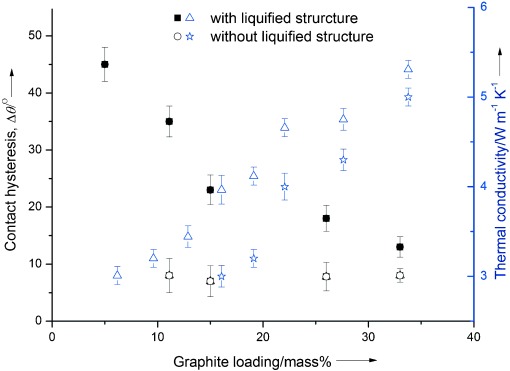
Contact angle hysteresis and thermal conductivity of composites as a function of graphite loading with and without molten-salt liquefied structure (MSLS) of MgO: for the composites, the mass ratio of salt to MgO is 1:1 for MSLS. ▵: thermal conductivity, ▪: contact angle hysteresis, Δ: thermal conductivity, ○: contact angle hysteresis. The error bars indicate standard deviation from repeated measurements.

As mentioned before, wettability is related to the contact angle. Figure 6 shows schematically the contact angle of molten salt on different surfaces, together with corresponding images of droplets obtained by a high-speed camera. The figure also illustrates the definition of the contact angle hysteresis as the difference between the advancing and receding contact angles of a moving droplet. Such a parameter characterizes the surface structure, with a very low value of the contact angle hysteresis indicating a nearly defect-free and smooth surface. Figure 7 shows the contact angle hysteresis with (Figure 6 B and Figure 6 C) and without (Figure 6 A) the MSLS of the supporting ceramic material (MgO) at different loadings of graphite (indicated by solid squares and empty circles). With MSLS of MgO as the substrate, the contact angle hysteresis is approximately 45° at a low graphite loading of 5 %. The hysteresis decreases rapidly with increasing loading of graphite to ca. 13° at a graphite loading of 33 %. However, the contact angle hysteresis is approximately 7.5° for the substrate without the MSLS of MgO and the graphite concentration does not seem to have any effect. The poor wettability of the molten salt on graphite as discussed earlier is considered as a possible reason for the relatively low contact angle hysteresis. Another possible reason is that, as illustrated in Figure 6 A, molten salt contained in the graphite can leak out as illustrated in Figure 4 B, forming a thin layer on the composite surface. When a molten salt droplet slides on the composite surface, it would be in contact with the thin layer rather than the actual substrate surface, leading to nearly constant value of the contact angle hysteresis. With the ceramic supporting material (Figure 6 B), the good wettability could drive the molten droplet to enter the pores of the substrate, leading to the pinning of the droplet and relatively high contact angle hysteresis on the composite. The addition of graphite into the MSLS of MgO substrate (Figure 6 C) is therefore expected to lead to a decrease in the contact angle hysteresis. The above analyses suggest that MSLS of MgO should play a significant role in retaining the molten salt within the structure. In the absence of the MSLS of MgO, the molten salt retreats from the pores as illustrated in Figure 6 A owing to poor wettability, leading to the formation of a porous structure. The more graphite is added, the more porous a structure is expected. These findings are consistent with the analyses given earlier.

Shown in Figure 7 also include the measured thermal conductivity of the composite substrates with and without the MSLS of MgO (empty triangles and stars in the figure, respectively). One can see that the thermal conductivity of the composite with the MSLS is higher than that without the MSLS. The porous structure due to the poor salt wettability on graphite is likely to be a reason as discussed above. Figure 7 also indicates that the difference between the thermal conductivities of the composite with and without the MSLS decreases with increasing graphite content in the composite. This could be explained by the fact that graphite has a very high thermal conductivity, which would exert more effect on the overall thermal conductivity at higher graphite contents.

### Thermal energy storage density

The thermal energy storage density of the composites, *Q* (J g^−1^), can be calculated by:


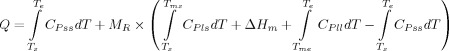
(2)

where *C_Pss_* is the heat capacity of the ceramic supporting material and graphite that do not go through phase change over the temperature range studied (J g^−1^ K^−1^), *C_Pls_* and *C_Pll_* denote respectively the heat capacity of the phase change material (PCM) in the solid phase and the liquid phase (J g^−1^ K^−1^), Δ*H_m_* is the latent heat of melting (J g^−1^), *T_s_* is the starting temperature (K), *T_ms_* and *T_me_* represent respectively the temperature of the PCM at which melting starts and ends (K), *T_e_* is the ending temperature (K), and *M_R_* is the mass fraction of the PCM in the composite. The above equation indicates that the total thermal energy storage density of PCM composites consists of both the latent heat of PCM and the sensible heat of all components within the composites. For a composite with a mass ratio of graphite/salt/MgO=0.25:1:1 working over a temperature range of 300–600 °C, the total energy density is approximately 525 kJ kg^−1^.

### Thermal characterization

**Figure 8:**
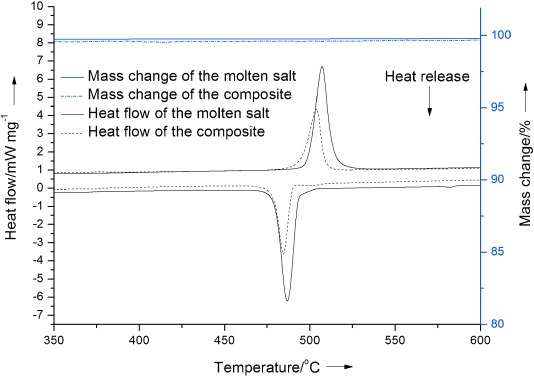
TGA–DSC curves for the salt and the composite containing graphite, salt, and MgO with a mass ratio of 0.05:1:1.

**Figure 9:**
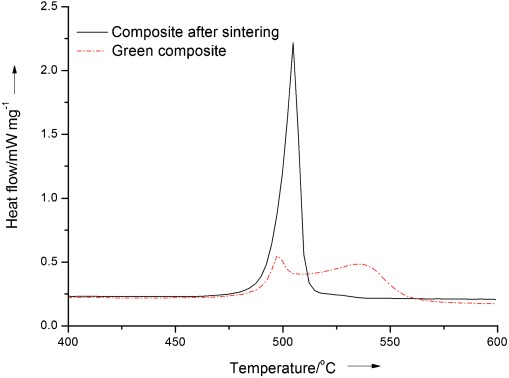
DSC curves for a green composite and a sintered composite containing graphite, salt, and MgO with a mass ratio of 0.25:1:1.

Figure 8 compares the thermal characterization of a salt and a graphite–salt–MgO composite with a mass ratio of 0.05:1:1 using combined DSC and TGA techniques. More thermophysical data obtained from the TGA-DSC analysis are listed in Table 1, which also contains statistical information on the confidence of the data. The TGA curves (top two lines in Figure 8) suggest high thermal stability for both the salt and the composite over the temperature range of 350–600 °C. The DSC curves during heating (middle curves in Figure 8) show that the salt starts to melt at 500.2 °C and that the melting process ends at about 512.1 °C, giving a latent heat of melting (Δ*H_m_*) of about 348.5 J g^−1^, whereas the composite melts over 496–507.1 °C with a latent heat of melting of about 178.3 J g^−1^. One can see that the start/end melting temperature of the composite shifts to the left by about 4–5 °C and the composite has a latent heat that is approximately 50 % that of the pure salt. This can be attributed to the addition of approximately 50 % of non-PCMs in the composite. The shift of the start/end of melting temperature of the composite (i.e., the PCM in the solid structure of the composite) may be associated with interactions between the PCM and the structure of the supporting material and graphite. Figure 9 shows melting DSC curves of green and sintered composites with the same composition. Unlike sintered composite, the DSC curve of the green composite shows two peaks, with one at approximately the same melting point as the pure salt (497 °C) and the other at a higher temperature, of about 540 °C. The whole melting process happens in a much wider temperature range. As illustrated earlier, these phenomena can be related to composite structure formation due to the pressure difference (Δ*P*) in the sintering process, during which non-PCM particles move and rearrange to balance Δ*P*. The sintered composite, on the other hand, has a rigid structure that makes the molten salt melt in a confined space. In the following, an explanation is put forward. We start with the following Gibbs–Thomson equation:[13]


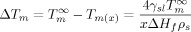
(3)

**Table 1:** Thermo-physical data of the molten salt and as-synthesized composite

Thermo-physical parameter	Pure molten salt (±95 % CI/SD)	Graphite–salt–MgO (±95 % CI/SD)
Start melting temperature, *T_ms_* [°C]	500.2 (3.97/1.6)	496.0 (1.3/0.5)
End melting temperature, *T_me_* [°C]	512.1 (2.0/0.8)	507.1 (3.4/1.4)
Latent heat of melting, Δ*H_m_* [J g^−1^]	348.5 (6.6/2.6)	178.3 (5.2/2.1)
Start freezing temperature, *T_fs_* [°C]	491.9 (2.9/1.2)	487.2 (1.7/0.7)
End freezing temperature, *T_fe_* [°C]	480.8 (2.8/1.2)	479.9 (3.3/1.3)
Latent heat of freezing Δ*H_f_* [J g^−1^]	−351.4 (5.3/2.15)	−183.5 (6.2/2.5)

where *T_m_*^*∞*^ is the bulk melting point of a solid material, *T*_*m(x)*_ is the melting point of a crystal with diameter *x*, γ_*sl*_ is the surface energy at the crystal-liquid interface, Δ*H_f_* is the bulk enthalpy of fusion per unit mass of the material, and *ρ_s_* is the density of the solid material. Equation (3) suggests that Δ*T_m_* be related to the liquid and solid properties of the material, and interfacial interactions between the two states. As a result, the Gibbs–Thomson equation has also been written as:


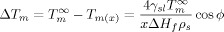
(4)

where *ϕ* is the contact angle between the liquid phase and the solid phase of the same material, which is often assumed to be a constant value of 180° in Equation (4). This may not be held for the phase change in confined regions. Recent studies on liquid-solid phase change in pores have indeed suggested deviation from the Gibbs–Thomson theory.[14] As illustrated in Figure 5, an equation analogous to Equation (1) (Figure 5 A) can be obtained for the case of coexisting solid and liquid phases in a pore (Figure 5 B) as follows:


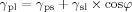
(5)

where γ is the interfacial tensions with the subscripts *p*, *l*, and *s* corresponding to the pore wall, liquid, and solid phases, respectively. Insertion of Equation (5) into Equation (4) gives:


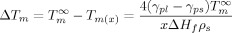
(6)

Equation (6) suggests dependence of Δ*T_m_* on the magnitudes of the pore wall-liquid and pore wall-solid interfacial tensions and hence provides an explanation for the melting temperature shift of the composite material with MSLS of the supporting material.

For the cooling/freezing process, DSC curves also show a temperature shift. Similar interpretations could be used, which will not be repeated here.

### Cyclic heating–cooling performance

**Figure 10:**
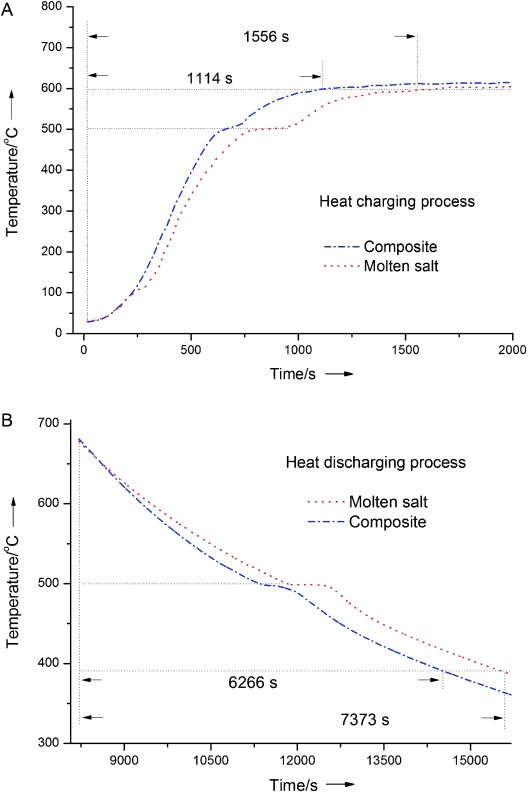
Sample temperatures as a function of time during (A) charging and (B) discharging for the pure salt and the composite containing graphite, salt, and MgO with a mass ratio of 0.25:1:1

Typical charging (heating) and discharging (cooling) processes of the molten salt and the composite are shown in Figure 10 A and B, respectively. The charging process shown in Figure 10 A was done under a constant heating (environmental) temperature of 750 °C. Figure 10 A seems to suggest that the final (equilibrium) temperatures of both the molten salt and the composite be around 610–620 °C. This is not the case because there are too many data points and not all are shown in the figure. The actual end point for the two samples (after around 7500 s) is approximately 680 °C, as can be seen from Figure 10 B (the starting temperature for the discharge process). Figure 10 A shows that the time dependence of the temperature of the composite is broadly similar to that of the molten salt except for the length of the constant temperature melting platform at about 500 °C due to the phase change and the heating rate. It takes 1114 s and 1556 s for the composite and the molten salt to reach 600 °C, respectively. These observations can be explained as follows: For a given mass under the given heat transfer condition, the more the phase change material in the sample, the more the heat is absorbed as the latent heat and the longer the time is needed to complete the melting process. If attention is paid to the time needed to reach 400 °C (before the phase change of the molten salt), the composite takes a shorter time than the molten salt does. This is because the composite has a higher thermal conductivity, as discussed before, and hence a higher heat transfer rate.

Figure 10 B shows the temperature as a function of time during the cooling (discharging) process. The phase change at around 500 °C is clearly seen in the figure, consistent with that shown in Figure 10 A. The Figure also shows that the time durations required for the composite to cool from 680 °C to 520 °C (before the phase change) and to 390 °C (after the phase change) are shorter than that for the molten salt, consistent with the charging process discussed above.

**Figure 11:**
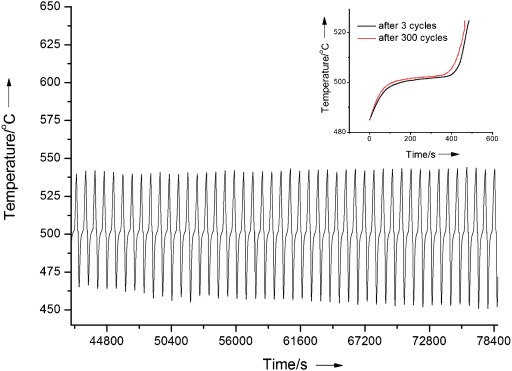
Sample temperatures as a function of time during cyclic heating and cooling experiments for composite containing graphite, salt and MgO with a mass ratio of 0.25:1:1. The inset compares the melting curves of the composite after 3 and 300 cycles.

Cyclic charge–discharge experiments have been done on the composite sample. Figure 11 shows the results in the form of sample temperature as a function of time for 46 cycles, which are taken from part of the data points from a 300-cycle test. The inset in Figure 11 shows a comparison of the melting curves of the composite after 3 and 300 cycles. No significant changes are seen in the duration of the melting process. This, together with the TGA and thermal conductivity measurements, suggests that the composite material have an excellent cyclic performance.

## Conclusions

The work reported herein addresses key challenges associated with the use of inorganic-salt-based medium- and high-temperature thermal energy storage materials. The focus is on the use of microstructured composite materials. Such materials consist of a molten-salt-based phase change material, a ceramic material, and a thermally conductive carbon material. Investigation into the microstructural development, chemical compatibility, thermal stability, thermal conductivity, and thermal energy storage behavior of the composite materials shows that the microstructures are able to hold the molten salt as well as improve the thermal conductivity. The microstructured materials are also shown to possess both a high energy density and a high power density.

## Experimental Section

### Materials and composite fabrication

The PCM used in this work was a eutectic salt of 49 % sodium carbonate (Na_2_CO_3_, Beijing Chemicals) and 51 % lithium carbonate (Li_2_CO_3_, Sinopharm Chemical Reagent Co. Ltd) with a melting temperature of ca. 497 °C and a solid-liquid phase change latent heat of ca. 351 J g^−1^. A ceramic material (MgO, Sinopharm Chemical Reagent Co., Ltd) and a carbon material (natural graphite flake, XiLong Chemicals) were chosen as the supporting material and thermal enhancing material, respectively. These materials were mixed according to a preset mass ratio and shaped into slabs by uniaxial cold compression (5 MPa) before sintering in an electric furnace using the following heating procedure: 25–400 °C at a heating rate of 5 °C min^−1^, 400–550 °C at 1 °C min^−1^, holding at 550 °C for 90 min. The cooling procedure was the reverse of the heating process.

### Morphology and microstructure characterization

The morphological and microstructural characterizations of the composites were carried out by using a scanning electron microscopy instrument (SEM, JSM-7100F). The crystal structure and the chemical compatibility of the composites were determined by X-ray diffraction (XRD, X′Pert PRO MPD) with a scanning angle of 5°–90° using Cu_Kα_ radiation.

### Wettability measurements

Wettability measurements were performed at 550 °C using the sessile-drop method in a tubular furnace under a nitrogen enviroment. Composite slabs with a size of 20 mm×20 mm×2 mm were used as substrates in the measurements, whereas a self-made funnel-like graphite divice was used to deliver molten salt droplets onto the substrate in a controlled manner. In a typical experiment, the substrate was inserted into the tubular furance first, followed by evacuating the furnace to −0.1 MPa at the room temperature. The furnace was then switched on to initiate the heating process under a nitrogen purge. When the furnace reached 550 °C, a droplet was delivered onto the substrate and evolution of the droplet shape was recorded by a high-speed digital video camera (Phantom v311), which, after data analyses, gave the surface wettability.

### Thermal characterization

A combined device consisting of differential scanning calorimetry (DSC) and a thermal gravimetric analysis (TGA) instruments (TGA-DSC, STA 449 F3 Jupiter) was used to evaluate the phase change behavior and the specific heat of the materials. In a typical measurement, about 5 mg composite material was used and the heating/cooling rate was set at 10 °C min^−1^. A laser flash apparatus (LFA 427, Germany) was used to measure composite thermal diffusivity (*α*). The thermal conductivity (*λ*) was calculated from the thermal diffusivity data through *λ=αρC_p_*, where *ρ* is the density and *C_p_* is the specific heat capacity. All measurements were repeated at least three times with the average taken for the analyses.

### Cyclic heating–cooling performance of composite materials

The cycling heating–cooling performance of the composite materials was evaluated using a self-constructed automated device consisting of a high-temperature region (750 °C) and a low-temperature region (20 °C) in a vertical configuration. Temperatures of the two regions and the sample were measured using Type K thermocouples linked to NI 9213 module. In a typical experiment, a sample was loaded into the high temperature region where it was heated up to go through the charging process. When the sample reached a temperature of 680 °C, it was automatically lowered into the low temperature region where it was cooled down to the room temperature to complete the discharging process. The sample was then moved upward to the high temperature region to complete the cycle. The process was then repeated for a preset number of 300 cycles.
